# Lipid-lowering Drugs as a Potential Cause of Psychiatric Adverse Reaction-A Case Report

**DOI:** 10.1192/j.eurpsy.2025.1251

**Published:** 2025-08-26

**Authors:** N. Rijavec

**Affiliations:** 1 University Psychiatric Clinic Ljubljana, Ljubljana, Slovenia

## Abstract

**Introduction:**

Cholesterol is important for the normal functioning of the central nervous system. However, its reduction by certain agents might be related to the psychiatric adverse reactions, which could vary in frequency, but some can be serious and even fatal, such as suicidality and severe aggression.

**Objectives:**

With this case report, we would like to draw attention to the possibility of serious psychiatric adverse reactions in patients receiving lipid-lowering drugs.

**Methods:**

Bibliographic search and description of a clinical case of a patient under follow-up in outpatient treatment.

**Results:**

A-60year old woman with generalized anxiety disorder and hyperlipidemia in a previously good remission reported increased anxiety, nervousness, insomnia and irritability on a regular check-up. There were no external stressors, except for an approaching retirement, which she was looking forward to. However, she forgot to mention that she was switched from fenofibrate to a pill consisting of a combination of rosuvastatin and ezetimibe. Her antianxiety medication needed to be increased and some cognitive behavior interventions were administered. However, her condition continued to deteriorate in the following months. She even developed suicidal ideation and verbal aggression. She refused to be hospitalized, while going to a trip with her husband. During the trip, she was not taking her new antilipemic medication and she never restarted it. After two days of discontinuation of the combination pill, her psychiatric condition gradually improved. At the following examination, she revealed the modification of her antilipemic treatment and the new medication could be identified as a cause of intensification of her anxiety. Subsequently, she was switched to atorvastatin (later on ezetimibe was added). She went into a full remission and she remained in it, while her psychiatric medications were reduced to the initial dosages.

**Conclusions:**

The treatment of the patient’s symptoms in our case report was directed towards reducing a substantial worsening of the anxiety disorder while lacking the information on the previous replacement of a lipid-lowering drug. Luckily it ended well. With our case report, we would like to re-emphasize the importance of caution and monitoring of side effects, education of patient and interdisciplinary professional communication when treating with lipid-lowering drugs, especially in patients with a known mental disorder.

**Disclosure of Interest:**

None Declared

